# Enhancing heat transfer at low temperatures by laser functionalization of the inner surface of metal pipes

**DOI:** 10.1038/s41598-024-53062-8

**Published:** 2024-01-31

**Authors:** Daniel Holder, Alexander Peter, Marc Kirsch, Sergio Cáceres, Rudolf Weber, Volkher Onuseit, Rudi Kulenovic, Jörg Starflinger, Thomas Graf

**Affiliations:** 1 Institut für Strahlwerkzeuge (IFSW), University of Stuttgart, Pfaffenwaldring 43, Stuttgart, Germany; 2 ARENA2036 Research Campus, Pfaffenwaldring 19, Stuttgart, Germany; 3 Institut für Kernenergetik und Energiesysteme (IKE), University of Stuttgart, Pfaffenwaldring 31, Stuttgart, Germany

**Keywords:** Applied optics, Energy infrastructure, Mechanical engineering

## Abstract

The latent heat transfer during vapour condensation in the condenser section of passive heat transport devices such as the two-phase closed thermosiphon is limited by film condensation. Dropwise condensation provides an increase of the heat transfer coefficient by up to one order of magnitude and can be achieved with a water-repellant surface. The inner surface of pipes made from stainless steel was functionalized by laser surface texturing with ultrashort laser pulses and subsequent storage in a liquid containing long-chained hydrocarbons. The pipes were separated into half-pipes by wire eroding to enable laser texturing of the inner surface, and were then joined by electron beam welding after laser texturing. As a result, superhydrophobic and water-repellent surfaces with a contact angle of 153° were obtained on the inner surface of the pipes with a length of up to 1 m. The functionalized pipes were used in the condenser section of a two-phase closed thermosiphon to demonstrate a heat transfer rate of 0.92 kW at 45 °C, which is approximately three times the heat transfer rate of 0.31 kW of a smooth reference pipe.

## Introduction

According to the latest IEA (International Energy Agency) reports, one of the underestimated factors influencing climate change and worldwide consumption of electricity is cooling^1,2^. One possible solution to this is the application of passive cooling systems. This generally includes systems that use the environment as the ultimate heat sink for cooling without active components. The applications are diverse and range from cooling systems for buildings^3–5^, the cooling of lithium batteries^6,7^ and photovoltaic cells^8,9^ to the fail-safe cooling of spent-fuel pools in a nuclear power plant^10–13^. One representative of these systems is the two-phase closed thermosiphon (TPCT)^14^. The operating principle of the TCPT is based on the evaporation of a working fluid at the heat source and the condensation of the resulting vapour at the heat sink^15,16^. One focus of optimisation currently is on improving the internal heat transfer on the condensation side of the TPCT^17–26^. In general, film condensation is predominant there. From an optimisation point of view, however, dropwise condensation with a heat transfer coefficient that is up to an order of magnitude larger than the one achieved with film condensation would be desirable^27,28^.

Dropwise condensation occurs when a surface is water repellent, i.e. hydrophobic or superhydrophobic, which causes water droplets to form on and roll off the surface^22,29–32^. Hydrophobic and superhydrophobic surfaces have a low surface energy and high contact angles exceeding 90° and 150°, respectively^33^. Cooling systems and heat exchangers are usually made from metals due to their high heat conductivity. However, the contact angle of water droplets on metallic surfaces is typically in the range of 45° < *θ* < 83° and thus metal surfaces are hydrophilic^34–36^. The contact angle of a substrate mainly depends on the surface topography and the surface chemistry^34,37–39^ and it can be modified by various surface functionalization processes^40–42^, such as laser structuring.

Laser machining with short or ultrashort laser pulses can be used to influence the surface topography by creation of geometries on a wide scale from several millimeters down to a few hundred nanometers: Laser micromachining can be used to create macroscopic features such as holes, trenches, patterns or complex geometries on the surface with dimensions in the range of several millimeters down to a few micrometers^43–46^. Laser texturing (also known as laser structuring) by direct laser interference patterning allows for the creation of microscopic and periodic features such as dimples or small holes on the surface with dimensions of a few micrometers^47–51^. Laser texturing by conventional scanning of the beam over the surface can be used for the creation of microscopic and periodic features such as spikes, holes, grooves and ripples on the surface with dimensions of a few micrometers down to few hundred nanometers, which are also known as laser-induced periodic surface structures (LIPSS)^34,36,52–58^. The latter approach with conventional scanning is especially interesting to shape the surface topography of large parts due to the low complexity of scanner-based setups and the potential for very high texturing rates^55,59^.

The type and the dimensions of the generated features during laser surface texturing by scanning mainly depend on the process parameters, such as the peak fluence^34,52–54,58^ the pulse repetition rate^54^, the scanning speed^36,60^, the line spacing^55^ and the number of pulses per spot^52,54,61^ or the accumulated fluence^56^ as well as the initial surface condition prior to texturing^36,57^.

Laser surface texturing not only changes the topography; it also influences the chemical composition of the surface. Measurements of the chemical composition of surfaces exposed to ambient atmosphere showed an increased amount of oxides on the surface directly after laser surface texturing. The oxidation causes a hydrophilic or superhydrophilic wetting behavior with contact angles down to below 10° immediately after laser texturing^34^. The wetting behavior changes over time after laser texturing to hydrophobic or even superhydrophobic^34–36,62^. The strongest increase of the contact angle occurs during the first days and weeks after structuring. Long-term studies of up to one year then showed a further but much smaller increase in the contact angle^62^. The change of the wetting behavior over time after laser texturing can be attributed to the accumulation of nonpolar hydrocarbons from the ambient air that are adsorbed on the textured surface^34,63,64^. The surface chemistry and thus the wetting behavior also depend on the cleaning agent if the surface has been cleaned after texturing^36,65^, and the atmosphere in which samples are stored^35,36,66^.

In previous work, most often only flat and small samples were laser-textured, characterized, and tested for the respective application. In the work presented in the following, the curved inner surface of straight pipes made from stainless steel with a length of up to 1 m was functionalized by laser surface texturing and subsequently stored in a liquid containing long-chain hydrocarbons to obtain water repellant surfaces with a static contact angle exceeding 150°. This allowed to enhance the heat transfer of a two-phase closed thermosiphon in the condenser section.

## Influence of the process parameters on the resulting surface topography and the achievable texturing rate

**Figure 1:**
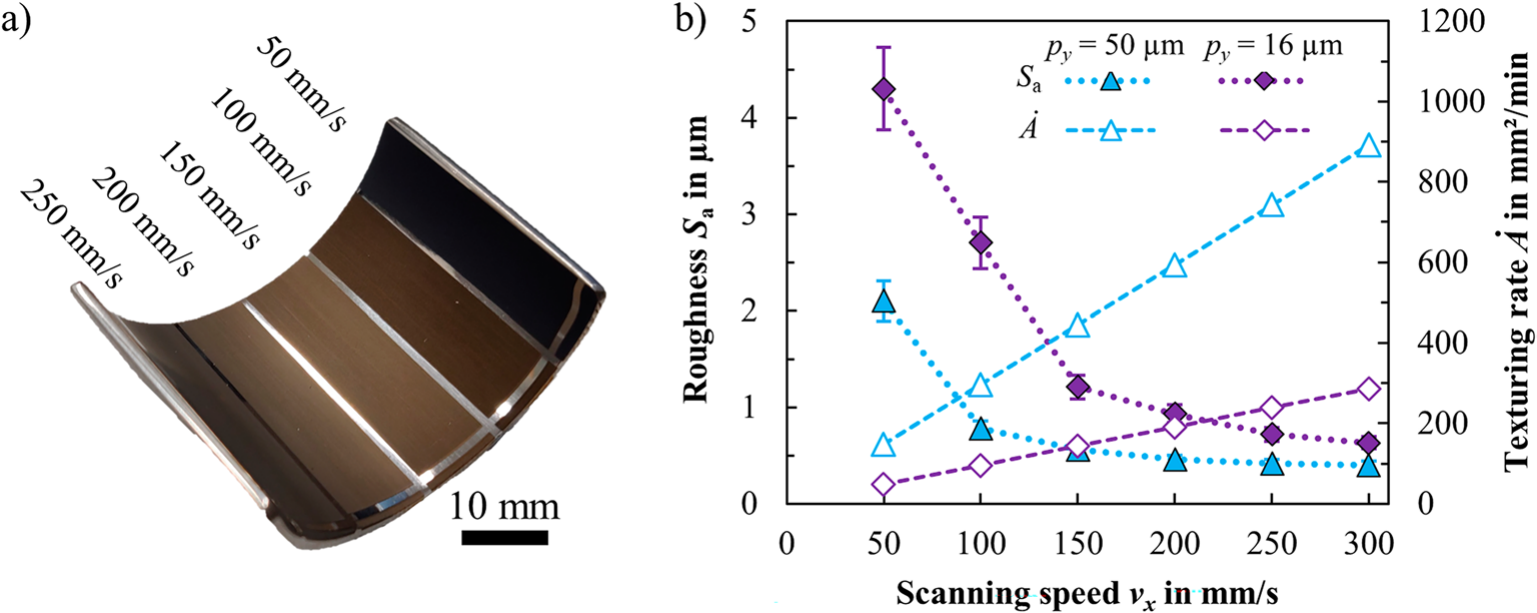
(**a**) Inner surface of the half-pipe after laser texturing with different scanning speeds and a line spacing *p*_*y*_ = 16 µm. (**b**) Measured roughness *S*_a_ and texturing rate *Ȧ* of laser-textured surfaces produced with different scanning speeds *v*_*x*_ and line spacings *p*_*y*_. The vertical error bars of the measured roughness represent the variation from randomly selected local measurements. *λ* = 1030 nm, *τ* = 8 ps, *d*_0_ = 77 ± 5 µm,* f*_rep_ = 300 kHz, *E*_P_ = 17.3 µJ, *n* = 1.

Figure 1a shows a photograph of an initially polished sample of the half-pipe textured with different scanning speeds.

The influence of the scanning speed *v*_*x*_ and the line spacing *p*_*y*_ on the surface structure generated on stainless steel was investigated using a constant pulse repetition rate of *f*_rep_ = 300 kHz, a constant peak fluence of *ϕ*_0_ = 0.7 J/cm^2^ and one scan per processed line (*n* = 1). The surface roughness

1 $$S_{{\text{a}}} = \frac{1}{M \cdot N}\sum\limits_{m = 1}^{M} {\sum\limits_{n = 1}^{N} {\left| {z\left( {x_{m} ,y_{n} } \right) - \left\langle z \right\rangle } \right|} } ,$$

where *z* is the height measured at the coordinates *x* and *y,* and where *M* and *N* are the number of measurements along the coordinates, respectively, was used to describe the surface topography in one value. The texturing rate

2 $$\dot{A} = \frac{{v_{x} \cdot p_{y} }}{n},$$

where *n* corresponds to the number of passes along the same scanning line, was used to describe the productivity of texturing. The surface roughness *S*_a_ (cf. Eq. (1) as well as the laser texturing rate *Ȧ* (cf. Eq. (2) are plotted as a function of the scanning speed *v*_*x*_ for different values of the line spacing *p*_*y*_ in Fig. 1b. Laser texturing leads to a roughening of the originally polished surface, which had a roughness of *S*_a_ ≈ 0.1 µm. Lower scanning speeds result in darker surfaces and a higher roughness *S*_a_ (cf. Fig. 1b, filled symbols). The rougher surface topography with decreasing scanning speed is caused by heat accumulation between subsequent pulses. The roughness significantly increases for scanning speeds below the „critical scanning speed “, which is described in^60^. Scanning with speeds below the critical scanning speed results in a steep increase of the roughness *S*_a_ > 1 µm, which occurs at *v*_*x*_ < 200 mm/s with *p*_*y*_ = 16 µm and at *v*_*x*_ < 100 mm/s with *p*_*y*_ = 50 µm. The roughness also occurs with a decrease of the line spacing *p*_y_, as shown in Fig. 1b. The higher roughness is linked to a lower texturing rate (cf. Fig. 1b, empty symbols) since low scanning speeds and small line spacings are required to produce rough structures.

**Figure 2:**
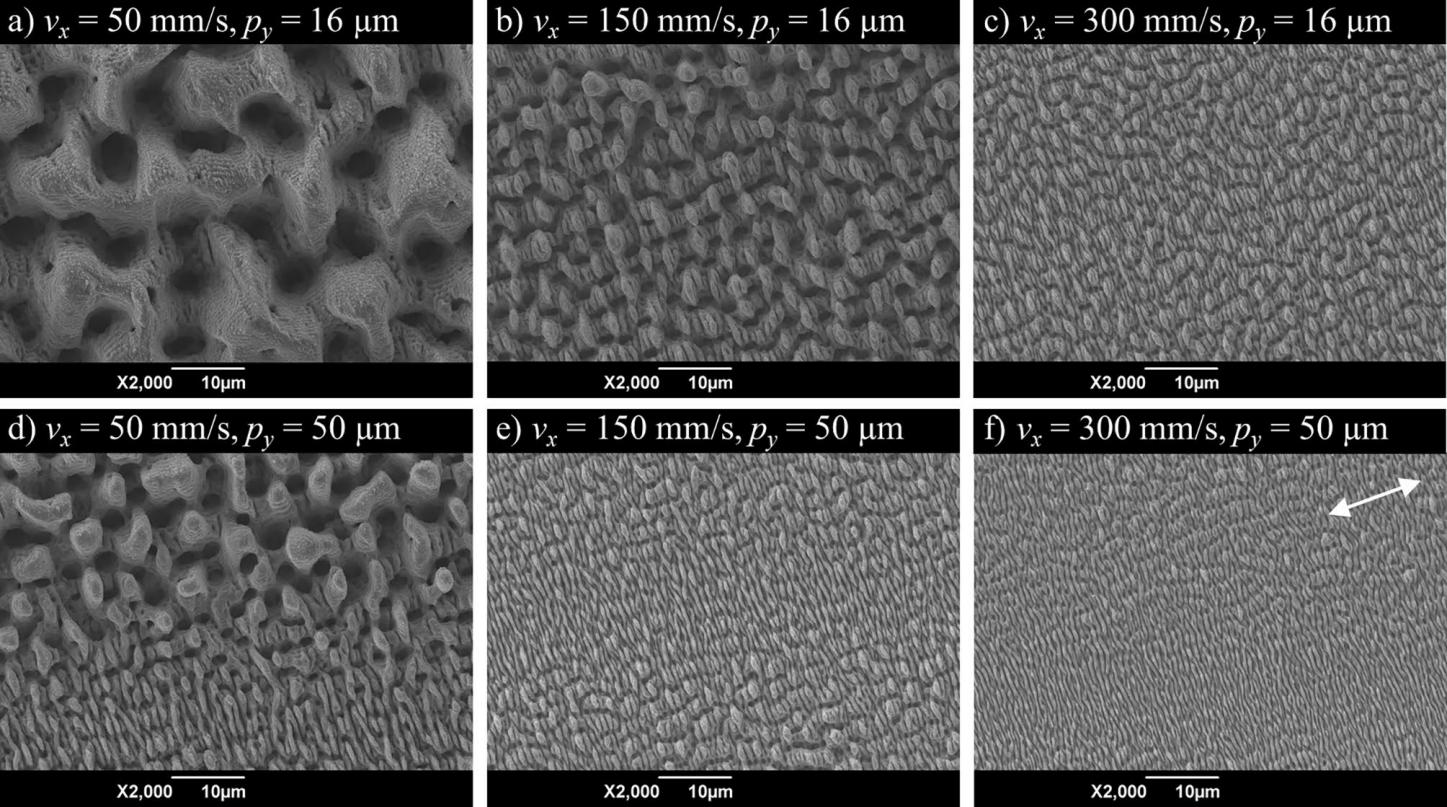
SEM images of laser-textured surfaces as measured after texturing with different scanning speeds *v*_*x*_ and line spacings *p*_*y*_. The white arrow indicates the orientation of the polarisation of the laser beam. *λ* = 1030 nm, *τ* = 8 ps, *d*_0_ = 77 ± 5 µm,* f*_rep_ = 300 kHz, *E*_P_ = 17.3 µJ.

Figure 2 shows SEM images of the surfaces resulting from surface texturing with different scanning speeds *v*_*x*_ and different line spacings *p*_*y*_.

The feature size and spatial period of laser-induced surface structures decrease with increasing scanning speed, which yields a reduced roughness *S*_a_ (cf. Fig. 1). Uniform LIPSS were achieved with the small line spacing of *p*_*y*_ = 16 µm (Fig. 2a–c). The hierarchical surface structures shown in Fig. 2a–c are usually referred to as “spikes and holes”, “rough microgrooves”, and “microgrooves and ripples”, respectively. Scanning with the larger line spacing of *p*_*y*_ = 50 µm led to an inhomogeneous surface topography with different kinds of LIPSS (Fig. 2d–f). The surface topographies shown in Fig. 2d–f consist of “spikes, holes and microgrooves”, “microgrooves and ripples”, and “ripples”, respectively.

The results demonstrate that the topography and the roughness of the laser-textured surfaces can be influenced by the processing parameters *v*_*x*_ and *p*_*y*_ and vary from fine ripple structures with a spatial period of about 1 µm and a roughness of *S*_a_ = 0.4 µm to rough spikes and holes with a spatial period of 15 µm and a roughness of *S*_a_ = 4.3 µm. The texturing rate achieved with the investigated process parameters ranges from *Ȧ* = 48 mm^2^/min for rough „spikes and holes “ (Fig. 2a) to *Ȧ* = 891 mm^2^/min for fine „ripples “ (Fig. 2f). Therefore, rough structures that require low scanning speeds and a small line spacing can be fabricated at significantly lower rates compared to fine structures that can be generated at high scanning speeds and a large line spacing.

## Influence of the surface topography and the storing conditions on the wettability of stainless steel

**Figure 3:**
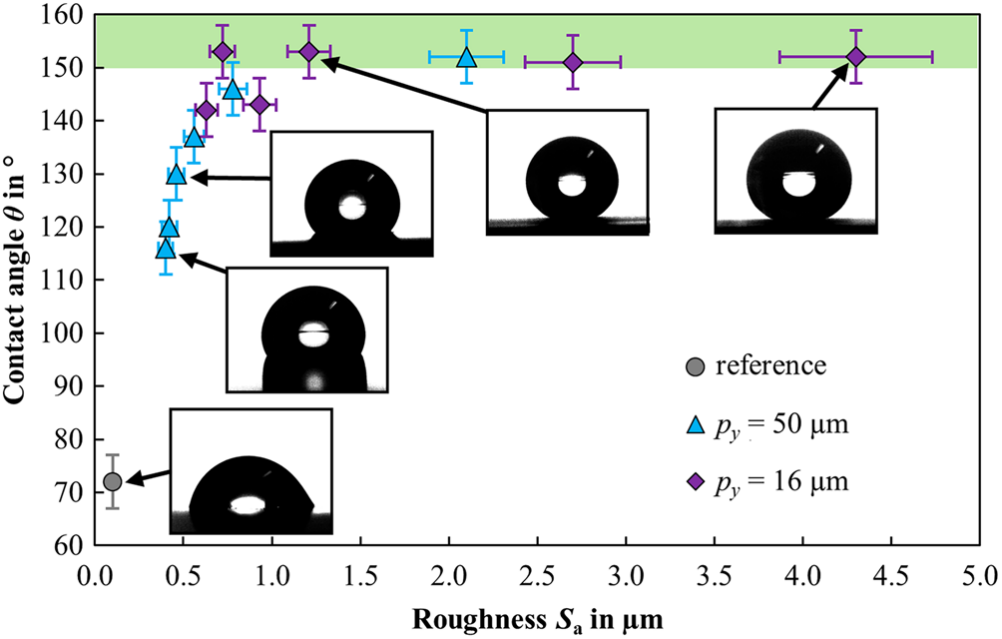
Contact angle *θ* as a function of the roughness *S*_a_ for differently textured surfaces. The surface without laser texturing is shown as a reference. The insets show the corresponding images of the droplets on the surface. The vertical and horizontal error bars represent the variation from randomly selected local measurements.

The wettability of the textured surfaces was measured approximately 30 days after laser texturing, as the static contact angle remains approximately constant after this duration^34,36^. The measured contact angles as a function of the surface roughness *S*_a_ as produced by laser texturing with different process parameters are shown in Fig. 3. Exemplary images of the droplets on the surface are shown in the insets.

A polished sample without a laser texture and with a roughness of *S*_a_ ≈ 0.1 µm was used as a reference and exhibited a hydrophilic surface with a contact angle *θ* = 72° (grey circle). Hydrophobic surfaces were obtained with laser-textured surfaces, whereby an increasing contact angle was observed with an increasing roughness of the laser-textured surfaces, up to the superhydrophobic range of *θ* > 150° (green area) at roughness values exceeding *S*_a_ ≈ 1.0 ± 0.3 µm. The contact angle remained almost constant in the range of 150° > *θ* < 160° for roughness values exceeding *S*_a_ ≈ 1.0 ± 0.3 µm up to the maximum investigated roughness of *S*_a_ = 4.3 µm. The data points in the range of *S*_a_ ≈ 1.0 ± 0.3 µm are of particular interest in this case to maximize the texturing rate for superhydrophobic surfaces, since the surfaces with higher roughness are created at lower texturing rates (cf. Fig. 1b) and yet do not allow an increase of the static contact angle.

**Figure 4:**
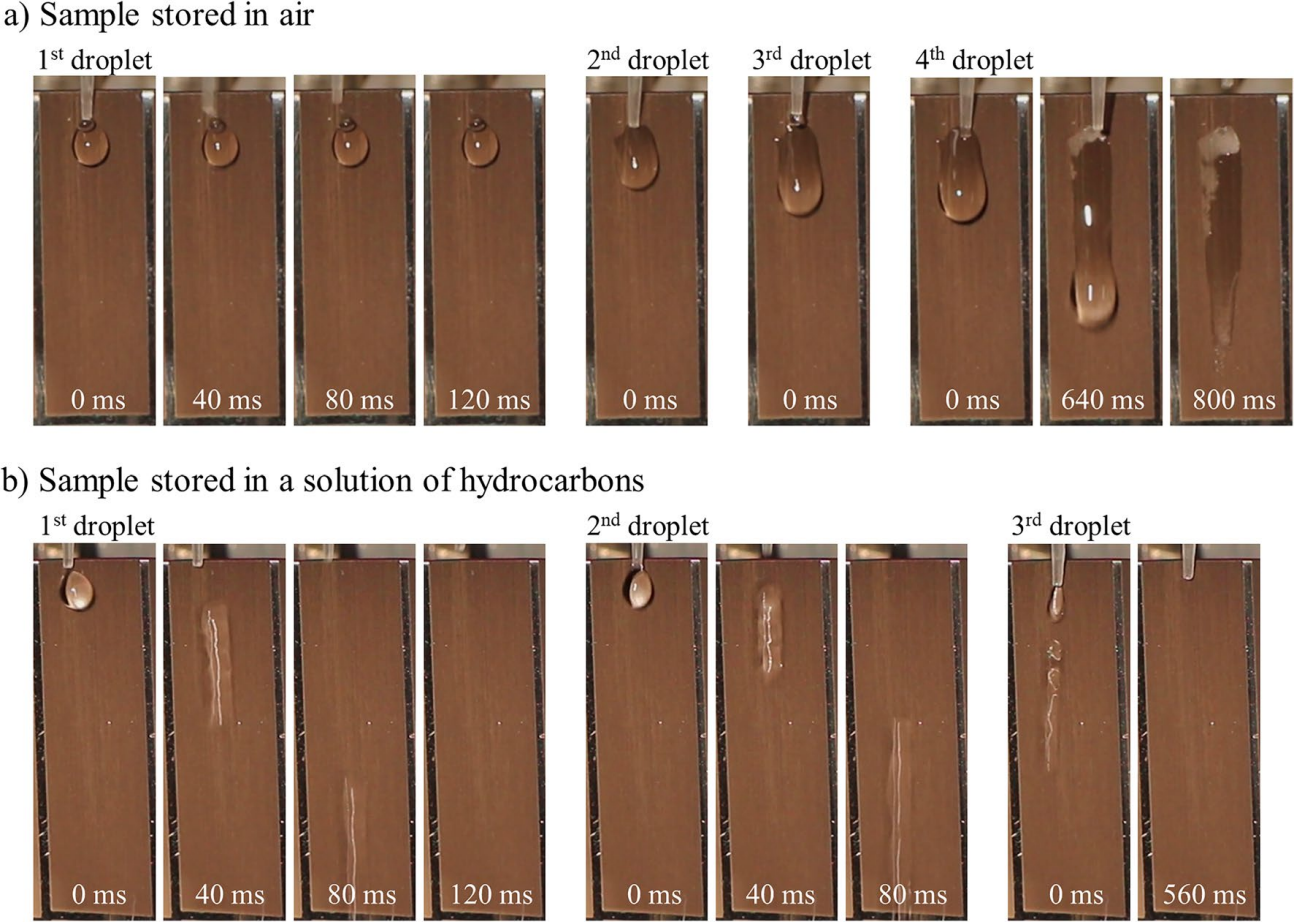
Images of the inclined laser-textured half-pipes stored in (**a**) air or (**b**) solutions of hydrocarbons showing the roll-off behavior of DI water droplets for different times after the application of the droplet.

Figure 4a shows images that were recorded during roll-off tests on a surface with “rough microgrooves” (*S*_a_ = 1.2 µm) and a static contact angle of *θ* = 153°, which was fabricated with a scanning speed of *v*_*x*_ = 150 mm/s and a line spacing of *p*_*y*_ = 16 µm. The samples were inclined approximately 80° in order to have an almost vertical orientation of the sample’s surface (corresponding to the TPCT application) but still be able to apply a water droplet on the top and characterize its roll-off behavior.

The application of the 1st water droplet led to the formation of a stationary droplet adhering to the surface, which is depicted in Fig. 4a for up to 120 ms after the application of the 1st droplet. The 1st water droplet still adhered to the surface at the application of the 2nd and 3rd water droplet, which were applied approximately 5 s and 10 s after the 1st water droplet. Each applied droplet increased the size of the droplet adhering to the surface. The force of gravity overcomes the force of the surface tension of the water droplet after the application of the 4th water droplet and caused the droplet to flow off (Fig. 4a), 640 ms), creating a remaining liquid film on the surface (Fig. 4a), 800 ms). This liquid film is disadvantageous for the operating performance of a TPCT, as a water film on the surface reduces the thermal conductivity of the pipe. A similar behavior regarding droplet formation, adherence on the surface, and creation of a liquid film was observed also for all the samples that were laser-textured and then stored in ambient air for 30 days. The reason for the film formation on all surfaces, including those with a contact angle of *θ* > 150°, is that a static contact angle measurement is not sufficient to fully describe the superhydrophobicity. This would require further dynamic contact angle measurements to determine the hysteresis and the sliding angle.

The laser texturing experiments were repeated with identical parameters on another sample, but with a different approach to subsequent sample storage in order to obtain superhydrophobic surfaces. The sample was laser-textured and stored in a solution of long-chained hydrocarbons to further modify the surface chemistry and enhance the water-repellency as shown in^36^. The measurements of the static contact angle of the surface with “rough microgrooves” after the storage in hydrocarbons again yielded *θ* > 150°, however, accompanied by a significantly improved roll-off behavior, as shown in the image sequence of Fig. 4b. Each of the three droplets rolled off the surface immediately, indicated by the motion blur in the images at 40 ms and 80 ms. The images taken 120 ms after the 1st water droplet and 560 ms after the 3 rd water droplet also show that the droplets rolled off without leaving any residue or liquid film on the surface. The deformation of the droplets indicates, however, that an increased repellency could be achieved with an improved surface coating, e.g. by using plasma-enhanced chemical vapor deposition (PECVD)^42^. Nevertheless, the results demonstrate that surfaces with strongly water-repellent properties can be achieved by fabrication of a rough surface texture and subsequent storage in a solution of long-chained hydrocarbons. The enhanced water-repellency was also obtained with laser-textured samples that were not mechanically polished before laser texturing of “rough microgrooves”, which indicates that this extra process step can potentially be omitted during the fabrication of functionalized steel surfaces.

## Application of laser-functionalized metal pipes to enhance the heat transfer in two-phase closed thermosiphons

The process parameters that result in a surface with “rough microgrooves” (*f*_rep_ = 300 kHz, *ϕ*_0_ = 0.7 J/cm^2^, *v*_*x*_ = 150 mm/s, *p*_*y*_ = 16 µm) were used to laser texture half-pipes made of stainless steel with a length of up to 1 m. The laser texturing of one half-pipe with a length of 1 m took about 6 h. The half-pipes were stored in the solution of long-chained hydrocarbons for three days after laser texturing to further enhance the water-repellency and then cleaned by storing in acetone for 15 min to remove any residues on the surface. Subsequently, the originally separated and then functionalized half-pipes were clamped in a setup of aluminum profiles and welded by electron beam welding (*Leibold-Heraeus, ESW-700-12*) with a line energy of 14 J/mm.

The aim of the laser texturing was to improve the internal heat transfer by forcing dropwise condensation inside the condenser of the TPCT. To prove the effect, tests with two different TPCTs were carried out at the boiling test rig of IKE, University of Stuttgart^67^. One of the TPCTs remained completely untreated (smooth reference pipe, *S*_a_ ≈ 0.5 µm) while the other one was functionalized by a superhydrophobic laser-textured surface (“rough microgrooves”, *S*_a_ ≈ 1.2 µm) in the condensation zone. Both TPCTs consisted of three zones, a 1 m long evaporation zone, a 1 m long adiabatic zone and a 1 m long condensation zone. For the construction of the TPCT, pipes made of AISI 304 with a total length of 3 m, a diameter of 38 mm (1.5″) and a thickness of 1.5 mm were used. Degassed deionized water was used as working fluid in both TPCTs.

**Figure 5:**
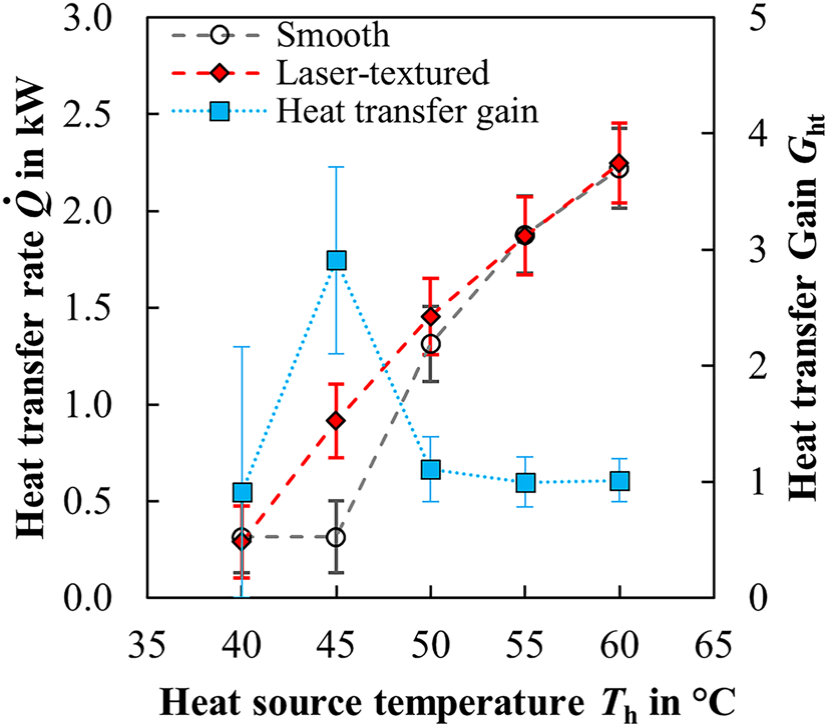
Comparison of the heat transfer rates achieved with an untreated smooth and laser-textured TPCT at different temperatures of the heat source. The heat transfer gain describes the ratio of the measured heat transfer of a laser textured half-pipe to a smooth half-pipe.

Figure 5 shows the comparison of the measurements on the laser-textured (red diamonds) and the untreated smooth reference pipe (white circles). The heat transfer rate on the condensation side is plotted as a function of the inlet temperature *T*_h_ of the heat source in the TPCT’s evaporation area. The heat transfer gain

3 $$G_{{{\text{ht}}}} = \frac{{\dot{Q}_{{{\text{las}}}} }}{{\dot{Q}_{{\text{ref}}} }},$$

describes the ratio of the measured heat transfer of a laser textured half-pipe to a smooth half-pipe. An increased performance of the laser-textured pipes is achieved for values of *G*_ht_ > 1. For *G*_ht_ ≈ 1, no significant increase in heat transfer is obtained. The heat transfer gain calculated from the measurements is plotted with blue squares in Fig. 5. All measurements were carried out at an inlet temperature of 20 °C of the heat sink in the TPCT’s condensation area and a working fluid filling level of 50% in the evaporator volume of the thermosiphon. It is noticeable that the laser-textured thermosiphon enters a steady-state operation at a lower temperature of the heat source than the one with the smooth reference pipe. Thus, an average heat transfer rate of *Q̇*_las_ = 0.92 kW (± 20% uncertainty) is achieved at an evaporation temperature of 45 °C, which is about three times the heat transfer rate of *Q̇*_ref_ = 0.31 kW (± 18% uncertainty) of the reference pipe.

This significant improvement of the heat transfer rate suggests the laser-textured surface indeed induces dropwise condensation in the condensation zone. At higher temperatures of the heat source, however, this effect evens out again, so that no distinct improvement is obtained by the laser-textured inner surface. The alignment of both curves suggests that the laser-textured surface is flooded at higher temperatures, which leads to a transition from the suspended Cassie–Baxter state to a pinned Wenzel state of the droplets and thus to a transition from dropwise condensation to film condensation^18,40,68^. An indication for this theory is the fact, that the surface subcooling, defined as the steam temperature against the wall temperature, increases due to the increased evaporation temperature from 0.1 °C at *T*_h_ = 40 °C to 2.2 °C at *T*_h_ = 45° to > 3.9 °C at *T*_h_ ≥ 50 °C. The results presented in^67^, which were measured in a larger temperature range at both the inlet of the evaporator and the condenser as well as with a higher filling level of the TPCT, showed that at high temperatures of 30 °C at the condenser inlet the heat transfer rate was enhanced from 11.5 to 13.1%. These results support the aforementioned assumption that the change in wetting state with increasing surface subcooling is a decisive factor influencing the improvement of the heat transfer rate. In the measurements with 30 °C condenser inlet temperature, the surface subcooling was significantly lower than in the measurements at 10 °C and 20 °C.

According to^69^, surpassing a critical ratio between the distances of the surface structures (correlation length) and the distance of nucleation for the condensation of droplets in the partial Cassie state is necessary for sustainable dropwise condensation on hierarchical surfaces. It is assumed that this criterion could be met, as the enhanced heat transfer at low temperatures could be maintained over the entire measurement campaign of 4 months with a total of over 500 operating hours. The repeatability of the experimental results demonstrates that a robust and durable water-repellency was achieved, which is necessary for the applicability of functionalized pipes in industrial applications.

## Conclusion

In conclusion, we have demonstrated an enhanced heat transfer at low temperatures induced by water-repellent laser-textured surfaces on the inside of metal pipes.

The surface topography on the inside of stainless-steel pipes was modified by laser texturing with ultrashort laser pulses, with surface roughness increasing and texturing rate decreasing with decreasing scanning speed and decreasing line spacing. A superhydrophobic and water-repellent surface with a contact angle of 153° was achieved by laser surface texturing of rough microgrooves and subsequent storage in a solution of long-chained hydrocarbons for three days. The integration of the functionalized pipes in the condenser section of a two-phase closed thermosiphon allowed to demonstrate a heat transfer rate of 0.92 kW at 45 °C and at 20 °C heat source respective heat sink temperature, which is approximately three times the heat transfer rate of 0.31 kW of a thermosiphon manufactured from an untreated smooth pipe used as a reference.

## Materials and methods

**Figure 6:**
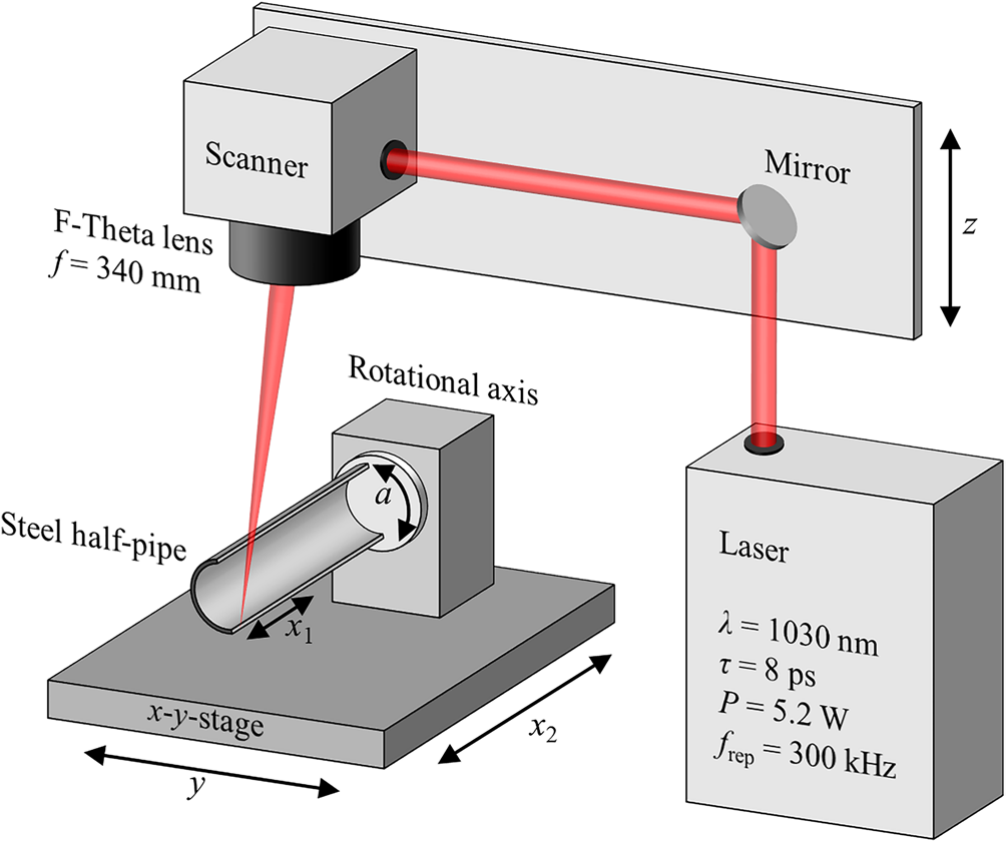
Experimental setup for laser texturing of the inner surface of half-pipes.

The pipes were made of the stainless steel AISI 304 with a length of up to 1 m, a diameter of 38 mm (1.5″) and a thickness of 1.5 mm. The pipes were longitudinally separated by wire eroding to gain access for laser surface texturing of the inner surface. The inner surfaces of the half-pipes were textured with ultrashort laser pulses using the experimental setup shown in Fig. 6.

An ultrafast laser system (*Trumpf*, *TruMicro 5050*) with a wavelength of *λ* = 1030 nm and a pulse duration of *τ* = 8 ps was used for laser texturing. The laser beam was linearly polarized and had a beam propagation factor of *M*^2^ < 1.3. The average laser power on the processed surface was 5.2 W, which corresponds to a pulse energy of *E*_P_ = 17.3 µJ at the pulse repetition rate of *f*_rep_ = 300 kHz. The laser beam was guided through a Galvanometer-scanner (*Scanlab*, *intelliSCAN 30*) and focused by an F-Theta lens with a focal length of *f* = 340 mm (*Sill Optics*). The beam diameter of the laser beam on the surface of the workpiece was measured to be *d*_0_ = 77 ± 5 µm. The scanning line of the Galvanometer-Scanner had maximum transversal dimension of 210 mm in *x*-direction (*x*_1_). Linear axes (*Trumpf, TLC5*) with a range of motion of up to 1 m (*x*_2_) were used for the positioning of the samples with an accuracy of ± 5 µm. This arrangement enabled a stitching of multiple scanning lines for uniform laser texturing of samples larger than one scanning line. The rotational axes (*isel, DSH-S*) allowed to rotate of the half-pipe (*a*-axis in Fig. 6) in order to maintain a perpendicular angle of incidence of the laser beam on the surface of the half-pipe and thus constant process parameters (e.g. absorbed fluence and pulse overlap).

Short half-pipes with a length of up to 50 mm were cut from the 1 m long half-pipes for the analysis of the surface and characterization of the wettability. The surfaces were investigated by scanning-electron-microscopy (SEM, *Jeol JSM-6490LV*) and the surface roughness was measured using a laser scanning microscope (LSM, *Keyence VK-9710-K*).

Prior to laser texturing, most of the half-pipes were mechanically polished on the inner surface to a surface roughness of *S*_a_ ≈ 0.1 µm to avoid the influence of the initial roughness on the generated surface topography. The short samples were textured along scanning lines with a length of up to 50 mm in *x*-direction (*x*_1_) with a unidirectional scanning strategy. The Sky-writing mode of the Galvanometer-scanner was used to ensure a constant scanning speed *v*_*x*_ during texturing. A two-dimensional texture was obtained on the half-pipes by rotating the *a*-axis, which resulted in a certain line spacing *p*_*y*_ that depended on the rotation speed.

The long half-pipes had to be subdivided into five parts of 200 mm length each, due to the limited field size of the Galvanometer-scanner. Each textured area was fabricated with a length of 200 mm in *x*-direction by scanning with the Galvanometer-scanner (*x*_1_) and with a width corresponding to the half inner circumference by rotation of the *a* axis. The tubes were then shifted by 200 mm in *x*-direction using the *x*_2_ axis of the *x–y-*stage for texturing the adjacent area. This stitching approach allows to functionalize large and heavy samples, as previously shown for an injection mold steel tool with a length of 500 mm and with a weight of 500 kg^45^.

The wettability of the laser-textured surfaces was characterized by the contact angle and the roll-off behavior of droplets on the surface. A camera-based measurement device (*DataPhysics, OCA 15 EC*) and the sessile drop method were used to determine the contact angle of deionized (DI) water on the samples. Droplets with volumes between 1 µℓ and 10 µℓ were supplied to the surface, whereas larger droplet volumes were required for very high contact angles *θ* > 150° in order to release the droplet from the pipette. The camera images of the droplets on the surface were taken approximately 3 s after the application of the droplets. The roll-off behavior of DI water droplets at room temperature (20 °C) was characterized with a self-built setup that uses a digital camera (*Canon, EOS 760D*). Multiple DI water droplets, each with a volume of 10 µℓ, were applied with a pipette while the camera recorded this process at a framerate of 25 fps. The samples, which were additionally chemically modified after laser texturing, were stored in a solution (*HPM, Sentos*) of long-chained hydrocarbons (n-alkanes, C10–C13, cyclic, < 2% aromatics) for three days directly after laser processing in order to further modify the surface chemistry and enhance the water-repellency. The samples were then cleaned by storing them in acetone for 5 min to remove any residues on the surface.

**Figure 7:**
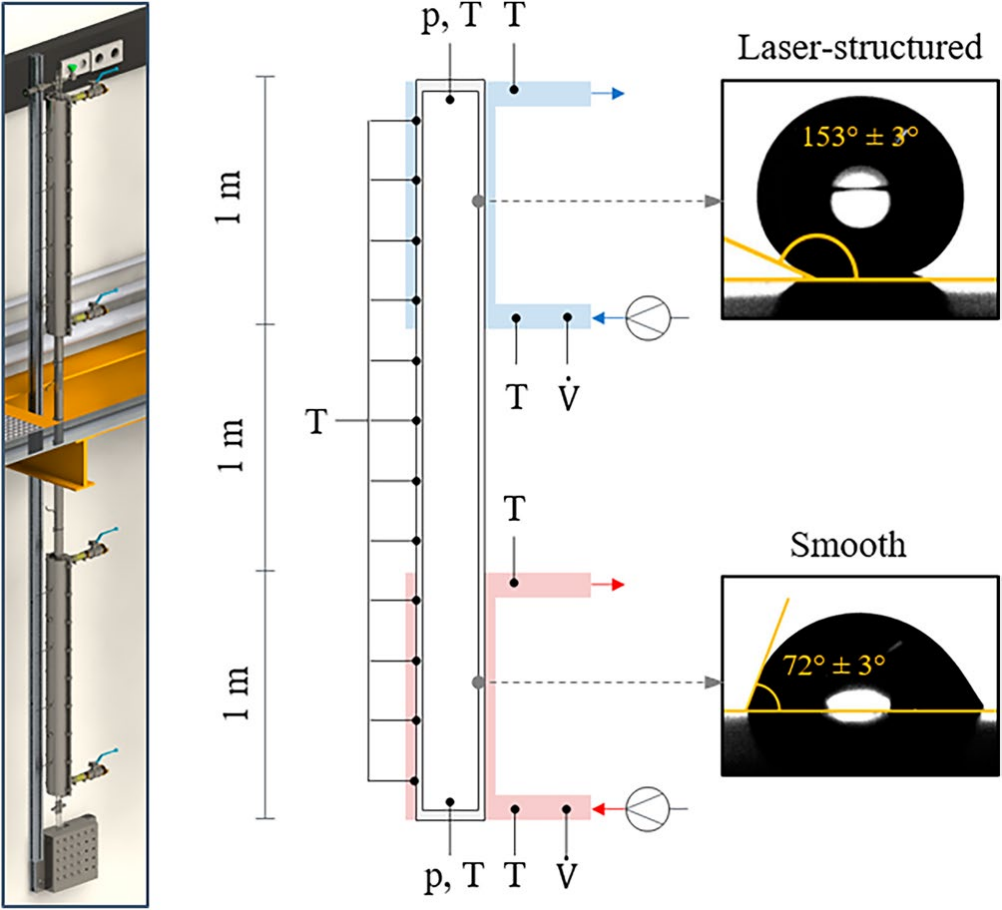
CAD drawing and scheme of the experimental arrangement with the positions for the measurement of temperature *T*, pressure *p* and flow rate *V̇*. The insets on the right show the determined contact angle of a water droplet on the laser-textured surfaces and on the smooth reference surface.

The experimental setup of the boiling test rig is schematically depicted in Fig. 7.

The identical measurement setup was applied to both TPCTs. All temperature sensors in the test rig were resistance temperature detectors PT-100. Twelve temperature sensors were mounted at equal distances of 250 mm on the outside of the TPCT to determine the surface temperature. Inside the TPCT there were four invasive temperature measuring points, two in the condensation zone and two in the evaporation zone. The absolute pressure was measured at the top and the bottom of the TPCT. Two process thermostats with water as working fluid were used for heating and cooling. Two identical semi-circular heat exchangers with an internal diameter of 47 mm were used to heat the evaporation zone and cool the condensation zone. The heat exchangers had eleven internal baffle plates guiding the working fluid of the thermostat circuits in a meandering flow path along the TPCT. For calorimetric measurements of the heat flow, a temperature measuring point was located both at the outlet and inlet of each heat exchanger. The volume flow rates of the two thermostat circuits were determined by means of ultrasonic flow meters. The flow rate of the temperature control circuit was adjusted via a bypass line. This also limited the fluctuations induced by the pump to 0.2 ℓ/s. The accuracies of the used measuring sensors are as follows: The absolute pressure sensors (*Omega, PAA-33x*) have an accuracy of ± 0.15% FS (full scale), all PT-100 temperature sensors have class A accuracy i.e. ± (0.15 + 0.002 T), and the measurement uncertainty of the volumetric flow meter is ± (0.7% RD + 0.7% FS), where RD stands for reading. The data acquisition was carried out by means of a data logger (*Keysight 34970A*), and the data processing was performed with a corresponding software (*Agilent VEE*). The entire measurement setup was insulated with an insulation material (*Armaflex XG*) having a thermal conductivity of 0.042 W/(m ∙ K). For the estimation of heat losses, the ambient temperature and the insulation temperature were also measured yielding heat losses of less than 2.5% in the investigated temperature range. Furthermore, each test campaign was repeated at least three times for every thermal boundary condition to check the reliability of the measured results.

## Data Availability

The datasets used and/or analyzed during the current study available from the corresponding author on reasonable request.
